# Academic Self-Efficacy and Academic Performance in Online Learning: A Mini Review

**DOI:** 10.3389/fpsyg.2018.02794

**Published:** 2019-01-22

**Authors:** Satoru Yokoyama

**Affiliations:** Department of Pharmacy, Chiba Institute of Science, Choshi, Japan

**Keywords:** academic self-efficacy, online learning, distance learning, e-learning, academic performance, academic achievement, learning outcome

## Abstract

Recently, online learning has been broadened to support learners’ learning processes. Attention has been given to academic self-efficacy (ASE) in educational psychology as an influential factor to enhance academic performance. Consequently, previous studies that have examined the relationship between ASE and online academic performance are reviewed and future directions discussed. Because there are limited findings to conduct a systematic meta-analysis on the relationship between ASE and academic performance in an online learning environment, a focused narrative review based on the previous findings are outlined. Finally, future directions, particularly in relation to similar and different aspects between a general learning and online learning environment are discussed.

## Introduction

Recently, online learning or distance education environments have been broadened to support learning processes in various domains and various levels of knowledge or proficiency. One of the themes that has attracted the most attention is how to maximize academic achievement or learning outcomes. While online learning outcomes has been reported to be influenced by ICT self-efficacy or other factors (e.g., So et al., 2012; Cussó-Calabuig et al., 2018), recent meta-analyses has recently attended to academic self-efficacy (ASE) (e.g., Robbins et al., 2004; Richardson et al., 2012; Honicke and Broadbent, 2016 for recent reviews about the relationship between ASE and academic performance).

In this article, previous findings on the relationship between ASE and academic performance in online learning setting are reviewed. Furthermore, the future directions of the relationship are discussed. Accordingly, the theoretical concept of self-efficacy (SE) is explained. The findings of the relationship between SE and learning/academic outcomes in general education/learning environments are briefly reviewed. At present, the limited findings do not permit a systematic meta-analysis of the relationship between ASE and academic performance in an online learning environment. Consequently, a focused narrative review based on the recent findings of the relationship is outlined. Finally, future directions particularly in relation to similar and different aspects between the general learning/educational environment and online learning environment are discussed.

## Self-Efficacy

The concept of general SE was originally proposed by Bandura in his social cognitive theory. SE may be defined as an individual’s belief in his or her ability to succeed in a specific situation or accomplish a specific task (e.g., Bandura, 1977, 1997, 2012). Although the concept of self-esteem is very similar, self-esteem involves an individual’s emotional evaluation of own value. In contrast, SE comprises an individual’s evaluation of own ability to achieve a goal or self-belief to do so. For example, in academic situation, it can be assumed that learners with high SE have higher motivation to learn, resulted in higher academic achievement, because those learners believe that they have an ability to achieve their goal. It is known that SE is influenced by gender, age, and domain. Huang (2012) conducted a meta-analysis and reported that ASE differs between gender, age, and also domains such as mathematics and social sciences.

From a theoretical perspective, SE can be strengthened through the experience of mastery, observing someone succeed, and social persuasion such as direct encouragement. In addition, physiological factors have been assumed to affect SE. For example, perceptions of pain, fatigue, and fear may have a marked, deleterious effect on SE. In a twin study, genetic factors explained 75% of the variation in SE (Waaktaar and Torgersen, 2013).

## The Influence of Ase on Academic Performance

To date, many previous studies have reported that a learner’s ASE is strongly associated with academic performance (e.g., Robbins et al., 2004; Richardson et al., 2012; Honicke and Broadbent, 2016). The results have consistently revealed that higher scores of ASE are more likely to result in higher levels of academic performance. Furthermore, Robbins et al. (2004) demonstrated that achievement motivation affects academic performance. Richardson et al. (2012) found that grade goals and effort regulation are strong factors in academic performance, similar to ASE. Honicke and Broadbent (2016) noted that effort regulation, deep processing strategies, and goal orientation have moderated the relationship between ASE and academic performance. As noted, goal-related aspects, that is, grade goals and goal orientation, and effort regulation have been found by two of three meta-analyses to be the strongest factors that influence academic performance other than ASE. Furthermore, although only a paucity of longitudinal studies has been conducted on the relationship between ASE and academic performance, the most recent meta-analysis has revealed that a higher ASE enhances academic performance longitudinally and vice versa (Talsma et al., 2018). In contrast, some of the studies have revealed no significant relationship between ASE and academic performance (e.g., Crippen et al., 2009; Cho and Shen, 2013; Gębka, 2014). Operationalization of ASE, timing of measurement, and cultural differences have been proposed as reasons (Honicke and Broadbent, 2016). Currently, it has been assumed that ASE is one of the most important factors or predictors for learners to achieve learning success. This may mean that if a student’s ASE is enhanced, the student may be able to achieve higher academic results.

## Methods

In this paper, previous studies were searched as follows. First of all, in Web of Science, the following searching parameters were used to search for previous studies: TS = [(“academic self-efficacy” OR “academic self efficacy”) AND (“academic achievement” OR “academic performance” OR “academic result” OR “learning outcome”) AND (e-learning OR “distance learning” OR “online learning” OR “distance education”)]. Only studies examining correlation or modeling between ASE and academic performance in online learning setting were selected manually. Also, in previous three meta-analysis studies examining the relationship between ASE and academic performance including all educational settings (i.e., Robbins et al., 2004; Richardson et al., 2012; Honicke and Broadbent, 2016), only studies in online learning setting were extracted from these reference lists. In addition, previous studies examining correlation or modeling between ASE and academic performance in online learning setting were extracted from these reference lists in the above extracted studies.

## Results

As a result, the following six studies were found: Lynch and Dembo (2004), Yukselturk and Bulut (2007), Kitsantas and Chow (2007), Crippen et al. (2009), Cho and Shen (2013), and Joo et al. (2013). The characteristics of these studies are presented in Table 1.

**Table 1:** The characteristics of six studies included in the current review.

	*N*	Age	Educational levels	Contents	Country
Lynch and Dembo, 2004	94	18–41	University	Marketing	United States
Yukselturk and Bulut, 2007	80	19∼	University	Programming	Turkey
Kitsantas and Chow, 2007	472	21.3	University including graduates	Educational psychology	United States
Crippen et al., 2009	176	No	University	Chemistry	United States
Cho and Shen, 2013	64	No	University	Gerontology	China
Joo et al., 2013	897	No	University	No description	Korea


### Effect of ASE on Academic Performance in Online Learning Setting

With regard to the effect of ASE on academic performance in an online learning environment, four studies revealed that ASE correlated with academic performance (Lynch and Dembo, 2004; Kitsantas and Chow, 2007; Yukselturk and Bulut, 2007; Joo et al., 2013), while two studies found no significant results (Crippen et al., 2009; Cho and Shen, 2013). Because all of the previous studies used the specialized content of a university/college course differently, the content cannot be used to explain the inconsistent results (see Table 1). Furthermore, cultural differences cannot be employed to explain inconsistent results because as shown in Table 1, because students from the United States and China participated in the two studies that yielded no significant results (Crippen et al., 2009; Cho and Shen, 2013), while United States, Turkish, and Korean students participated in the studies that revealed significant findings (Lynch and Dembo, 2004; Kitsantas and Chow, 2007; Yukselturk and Bulut, 2007; Joo et al., 2013). The participants in all the studies had similar educational levels as they were university/college students (see Table 1).

Neither this study nor Honicke and Broadbent (2016) discovered a convincing single explanation for such differing results, but there may be one possibility. The 53 studies analyzed by Honicke and Broadbent (2016) include all learning settings, and only six (11.3%) showed no significant correlation between ASE and academic performance. Two of the six examined studies (33%) show no significant correlation, and the ratio was larger for online classes than traditional classrooms. The larger ratio may reflect factors other than ASE, such as learners’ attitudes toward online instruction or familiarity with online learning devices (Cussó-Calabuig et al., 2018).

Studies reporting significant correlations (Richardson et al., 2012; Honicke and Broadbent, 2016) have reported differing trends. For example, in a traditional learning environment, grade goals, goal orientation, and effort regulation have the strongest influences, other than self-efficacy, on learning outcomes. Task value correlates with online learning outcomes in two of the six studies. This difference might be ascribed to students’ characteristics and differences between classroom and online learning. For instance, online instruction is task-based and perhaps motivates students who have a task orientation.

### Potential Factors That Correlate With Online Learning Outcomes

Previous studies have also examined various factors for academic performance in online learning setting other than ASE. For example, Yukselturk and Bulut (2007) and Joo et al. (2013) found that task value correlated with online learning outcomes. Furthermore, Yukselturk and Bulut (2007) and Crippen et al. (2009) revealed mastery-approach goal and intrinsic motivation as factors, respectively. These two factors are assumed to be related to the motivation to learn. In addition, verbal ability (Lynch and Dembo, 2004), educational level, help-seeking, threats (Kitsantas and Chow, 2007), self-regulated learning strategies, cognitive strategy use (Yukselturk and Bulut, 2007), login time, effort regulation (Cho and Shen, 2013), satisfaction, and persistence (Joo et al., 2013) were reported to be correlated with online learning outcomes.

### Factors That Were Tested but Not Significantly Correlated With Online Learning Outcomes

Previous studies have also revealed several factors, which were not significantly correlated with online learning outcomes. Although various factors did not have a strong significant correlation, extrinsic motivation was found not to be significantly correlated in two of the six studies (Yukselturk and Bulut, 2007; Cho and Shen, 2013). In addition, two of the six studies demonstrated that intrinsic motivation-related factors were not significant (Lynch and Dembo, 2004; Cho and Shen, 2013). The findings for this intrinsic motivation-related factor are contradictory because a mastery-approach goal and intrinsic motivation were found to be significant in other studies as described above (Yukselturk and Bulut, 2007; Crippen et al., 2009).

## Pedagogical Implications and Directions for Future Research

Previous findings on the relationship between ASE and academic performance in online learning setting have been summarized in this review article. Presently, while there are insufficient findings to conduct a meta-analysis, it can be postulated that ASE tended to correlate with academic performance in online learning environment, similar to a general learning environment. However, different trends were also found between online and general learning environments. The characteristics specific to online learning environment may affect connections between ASE and academic performance.

In particular, the current results suggest that students, teachers, and parents may have to pay attention to the following two points. First, since a familiarity with online learning devices may affect the relationship between ASE and academic performance in online learning settings, those who are not good at using online learning devices may not achieve high enough academic success in an online learning setting. Second, since task values are closely related to the relationship between ASE and academic performance, students, teachers, and parents may need to choose the online learning software they believe will have the most valuable content and/or tasks for students.

For researchers, it is recommended that future studies examine the relationship between ASE and academic performance in online learning setting and determine the influential factors in the relationship. In particular, because of possible differences between a general learning environment and online learning environment, it is necessary to examine what causes the differences between the two. Subsequently, based on these findings, future studies should add more experimental findings in online learning setting, and conduct a meta-analysis to clarify the relationship between ASE and academic performance in online learning setting systematically as well as its other influential factors, such as learner’s familiarity with, attitudes toward, and competence with, online learning devices.

## Author Contributions

The author confirms being the sole contributor of this work and has approved it for publication.

## Conflict of Interest Statement

The author declares that the research was conducted in the absence of any commercial or financial relationships that could be construed as a potential conflict of interest.
